# Elevated Neutrophil-to-Lymphocyte Ratio Is Associated with Severe Asthma Exacerbation in Children

**DOI:** 10.3390/jcm12093312

**Published:** 2023-05-06

**Authors:** Noga Arwas, Sharon Uzan Shvartzman, Aviv Goldbart, Romi Bari, Itai Hazan, Amir Horev, Inbal Golan Tripto

**Affiliations:** 1 Department of Pediatrics, Soroka University Medical Center, Beer-Sheva 8410101, Israel; noga.arwas@gmail.com (N.A.);; 2 Faculty of Health Sciences, Ben-Gurion University of the Negev, Beer-Sheva 8410101, Israel; uzansharon1@gmail.com (S.U.S.); romiba6@gmail.com (R.B.); itaihazan@gmail.com (I.H.); horev8@gmail.com (A.H.); 3 Pediatric Pulmonary Unit, Soroka University Medical Center, Beer-Sheva 8410101, Israel; 4 Clinical Research Center, Soroka University Medical Center, Beer-Sheva 8410101, Israel; 5 Pediatric Dermatology Service, Soroka University Medical Center, Beer-Sheva 8410101, Israel

**Keywords:** neutrophil-to-lymphocyte ratio, asthma, asthma exacerbation, emergency department, children, chronic inflammation

## Abstract

Asthma is the most common chronic respiratory disease in children. The neutrophil-to-lymphocyte ratio (NLR) is a marker of a chronic inflammatory state; however, data on the association of NLR with acute asthma exacerbations in children is lacking. In this cross-sectional study, between 2016 and 2021, children aged 2–18 years who were referred to the emergency department (ED) due to asthma exacerbation, were included. NLR, calculated from complete blood count upon arrival, was assessed as a continuous variable and was classified into four groups according to quartiles. The association between severity parameters and NLR quartiles was examined. A total of 831 ED visits for asthma exacerbation were included in the study. The median NLR was 1.6, 3.8, 6.7, and 12.9 in quartiles 1–4, respectively (*p* < 0.001). Demographic parameters, background diseases, and chronic medications were similar between the quartiles. Higher heart rate, body temperature, systolic blood pressure, and respiratory rate were observed in the higher NLR quartiles, as well as lower oxygen saturation. Higher urgency scale and higher rates of intravenous magnesium sulfate were observed in the higher NLR quartiles, with higher admission rates and prolonged hospitalizations. In summary, NLR upon admission is associated with the severity of asthma exacerbation and higher chances of hospitalization among children in the ED.

## 1. Introduction

Asthma is the most common chronic respiratory disease in children. It is characterized by chronic airway inflammation and hyper-responsiveness. Asthma exacerbations are well-known complications, impairing patients’ quality of life and causing substantial healthcare costs [1,2]. In a US study conducted between 2010 and 2015, asthma exacerbations accounted for 3% of emergency department (ED) visits and 6% of hospitalizations among children aged 5–17 years [3]. Among the common triggers for acute exacerbations in the pediatric population are respiratory viral infections and exposure to allergens [4]. 

Assessing the risk for a severe exacerbation at the initial evaluation in an asthma patient may be challenging. Some of the predictors previously reported include spirometry measurements, such as forced expiratory volume in the first second (FEV1), number of exacerbations in the previous year, and patients’ self-report on asthma control [5,6]. Several pediatric asthma scores are available to help classify the severity of exacerbation, including the Preschool Respiratory Assessment Measure (PRAM), the Pediatric Asthma Severity Score (PASS), and the Pediatric Asthma Score (PAS). Most scores assess different clinical-based parameters such as signs of increased work of breathing and accessory muscle use, air entry, and wheezing. Some include respiratory rate and oxygen saturation in addition to clinical observation [7]. In the acute ED setting, there is high variability among different centers in the management of acute exacerbation and admission criteria. The proportion admitted to the hospital ranges from 1.2–53%, according to different reports [8], and there is a non-negligible rate of relapse among discharged patients [9]. To date, there is no single validated model in determining the need for hospitalization in acute asthma exacerbation. Gorelick et al. suggested a model that can distinguish the patients that can be discharged from the ED from those needing further care, based on a clinical score, and the number of albuterol treatments given in the ED. Nevertheless, their model did not determine the need for a short stay versus more intensive inpatient-level care [8]. 

In the pathogenesis of asthma, airway inflammation is assumed to include two main subtypes, either induced by interleukin (IL)-5-mediated eosinophilic inflammation or an IL-8-mediated neutrophilic inflammation [10,11]. The role of systemic inflammation in asthma has not been well understood. An elevated level of circulating pro-inflammatory factors may be found in asthmatic patients. These include an increase in immune cells such as neutrophils and IL-6 and tumor necrosis factor (TNF)-α, which stimulates the production of acute-phase proteins [12,13]. Based on sputum eosinophil and neutrophil proportions [14], airway inflammatory patterns may represent different asthma phenotypes, with varying risks for exacerbations and response to treatment [15]. An increased neutrophil count in the sputum has been associated with steroid-refractory asthma and was negatively correlated with airflow obstruction parameters [14,16,17]. Peripheral blood counts may be of value in distinguishing between asthma phenotypes through correlation with sputum cell counts. Blood eosinophils, eosinophil-to-lymphocyte ratio (ELR), eosinophil-to-neutrophil ratio (ENR), and eosinophil-to-monocyte ratio (EMR) can predict eosinophilic asthma in adult patients [18]. 

The neutrophil-to-lymphocyte ratio (NLR) is a marker of a chronic inflammatory state. Previous data suggest its use as a prognostic factor indicating an accelerated inflammatory response in several diseases [19]. NLR was described in evaluating obstructive sleep apnea, allergic rhinitis, and chronic obstructive pulmonary disease (COPD) [20,21]. Moosman et al. reported age- and sex-specific pediatric reference values for the NLR and other blood count-derived biomarkers from 60,682 patients, from birth to 18 years of age. They found that the major changes in laboratory analysis from the neonatal period to adolescence were characterized by higher values directly after birth, which gradually decreased during the first two years of life. Another peak was observed in adolescence, with little change in most of the childhood years. No significant differences were found between boys and girls. It was also concluded that NLR was significantly increased in several diseases with inflammatory components, such as appendicitis; 65.7% of patients with appendicitis showed a higher NLR than the calculated upper reference limit. Regarding the respiratory system, they found that 22.7% of patients suffering from cystic fibrosis had pathological NLR values. In patients with asthma, NLR was also significantly increased; 20.6% of asthmatic patients presented with NLR > 97.5th percentile [22]. 

Measuring NLR is widely available, fast, and inexpensive, suiting both hospital and outpatient settings [23], though the clinical use of NLR in asthma patients is still debated. Imtiaz et al. did not find a relationship between asthma and NLR [24]. However, Zhang et al. reported an elevated NLR in adult patients with neutrophilic asthma [18]. In a study assessing the association of blood cell count parameters with severe asthma exacerbations, NLR was correlated with an increased risk for a severe exacerbation within the next year, with a predictor cutoff of 2.1 [23]. Multiple studies describe NLR in adult asthma exacerbation; however, there are only a few studies on children. Several previous studies evaluated NLR in correlation to a single parameter of hospitalized versus non-hospitalized pediatric patients [25]. Dogru et al. reported that NLR was higher by 17% in asthmatic children than in non-asthmatic controls [26]. Zhu et al. reported a combined score of C-Reactive-Protein and NLR levels, serving as a marker for differentiation between children with exacerbated asthma and healthy children [27]. In another recent study that included 89 children with asthma, the combination of NLR, alanine aminotransferase ratio, and NLR–albumin ratio was suggested as a clinical biomarker for asthma exacerbation in children [28]. 

To date, NLR is not routinely used to evaluate acute asthma exacerbation in children. Therefore, we aim to investigate the association of NLR with severity assessment and management of acute asthma exacerbation in children. 

## 2. Materials and Methods

This is a cross-sectional study examining the NLR and its association with acute asthma exacerbation in the Soroka University Medical Center, a single tertiary center in southern Israel. We identified children who were diagnosed with asthma as a background disease and were referred to the ED due to asthma exacerbation. We collected data on their medical history, laboratory results, and vital signs during their ED visit and hospitalization. 

### 2.1. Patients

A total of 715 pediatric patients aged 2–18 who presented during 813 ED visits for asthma exacerbation, between 2016 and 2021, were included in the study. All patients were presented with asthma-related symptoms (e.g., shortness of breath, wheezing, tachypnea), and underwent a complete blood count (CBC) upon arrival. In order to enhance precision, we included only the patients treated during the visit with anti-asthmatic medications. Children with respiratory symptoms that could be explained by factors other than asthma (e.g., suspected bacterial infection, chronic inflammatory diseases) were excluded from the cohort. We collected clinical, radiological, and laboratory data on each patient using electronic health record data. Data collected included: demographic data, chronic diagnoses, chronic medication purchases, vital signs on admission, radiological findings on chest X-ray (interpreted by either a pediatric radiologist or a pediatric pulmonologist), treatment at the ED, steroid use up to seven days prior to the ED visit, ED urgency scale based on the Canadian Triage Assessment Score (a clinical scale between 1–5, given at triage, according to initial patient assessment, with 1 being the most urgent and 5 the least urgent) [29], physical examination findings, CBC parameters, ED treatment, the decision of admission or discharge, treatment at the pediatric ward, pediatric intensive care unit (PICU) admissions, and length of hospitalization stay (LOS). Chronic diagnoses were extracted from the medical records, according to the ICD-9. The chronic diseases were categorized according to the involved system: endocrine disorders such as hypothyroidism and diabetes mellitus, cardiac diseases such as atrial septal defect and ventricular septal defect, metabolic diseases such as Niemann–Pick and mucopolysaccharidoses, developmental diseases such as cerebral palsy and autistic spectrum disorders, neuromuscular diseases such as Duchenne muscular dystrophy and myasthenia gravis, and gastrointestinal diseases such as inguinal hernia and short bowel syndrome. Pulmonary hypertension (PHT) was defined by echocardiographic diagnosis, that is, based on the task force of the European Society of Cardiology and the European Respiratory Society definitions of estimated systolic PAP ≥ 40 mmHg by Echo [30]. Bronchopulmonary dysplasia (BPD) was defined as a chronic lung disease of premature infants (<32 weeks gestational age), characterized by persistent parenchymal lung disease with radiographic confirmation, that requires oxygen or other respiratory support for ≥3 consecutive days, at 36 weeks postmenstrual age [31,32,33]. The study received the approval of the local institutional ethics committee (No. 184-20).

### 2.2. NLR

NLR was calculated by dividing the absolute neutrophil count by the absolute lymphocyte count in the blood count measured during the ED visit. Subsequently, the NLR was assessed as a continuous variable and was classified into four groups according to quartiles. In this manner, the cohort was divided into four quartiles [34,35], with NLR quartiles 1, 2, 3, and 4 representing 0–24.99%, 25–49.99%, 50–74.99%, and 75–100%, respectively.

### 2.3. Statistical Analysis 

Comparisons of demographic, clinical, and radiological parameters, as well as admissions outcomes, were made using appropriate univariate analyses. Continuous variables are reported as mean ± standard deviation (SD), median and interquartile range (IQR) for both normal and abnormal distribution. Categorical variables are presented as percentages. Comparisons were made with the appropriate statistical test. Continuous variables were compared with an ANOVA test, ordinal variables were compared with the Kruskal–Wallis test, and nominal variables were compared using chi-square analysis.

**Figure 1:**
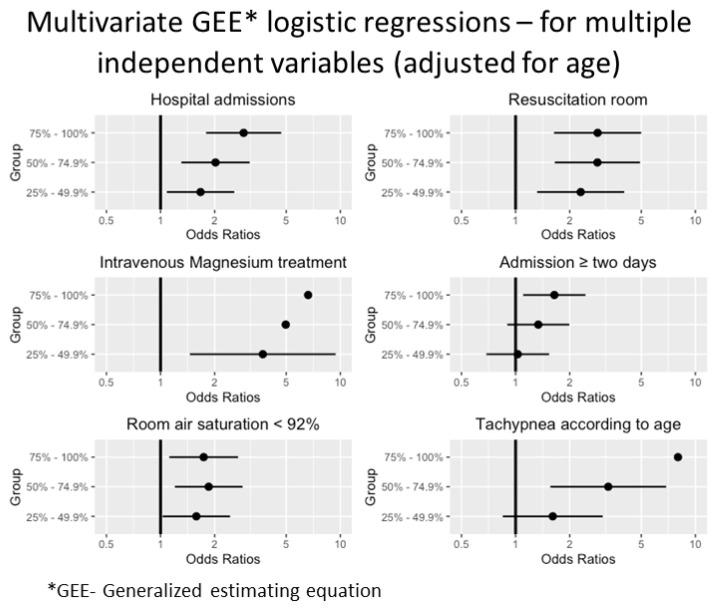
Multivariate GEE* logistic regressions for multiple independent variables, adjusted for age.

Given 117 repeated visits, we conducted several generalized estimating equations (GEE), with a logistic distribution. We chose the following variables as dependent variables: hospital admissions, the need for a resuscitation room, intravenous magnesium sulfate, admission ≥ two days, room air saturation < 92% [36], and tachypnea according to age. NLR quartiles were defined as primary independent variables. All regressions were adjusted for age. The results of the GEE models are shown schematically in Figure 1. The results are also shown in Supplementary Table S1 as an odds ratio (OR), 95% confidence interval (CI), and *p*-value.

A two-sided *p* value < 0.05 was considered statistically significant for all statistical tests. Reported *p*-values were rounded to two decimal places. All statistical analyses were performed using R statistical software version 4.05.

## 3. Results 

A total of 831 ED visits for asthma exacerbation were included in the study during 2016–2021. Of all visits during the study period (5 years): 638, 59, 8, 5, 3, and 2 children visited 1, 2, 3, 4, 5, and 8 times, respectively. The median NLR was 1.6, 3.8, 6.7, and 12.9 in quartiles 1, 2, 3, and 4, respectively (*p* < 0.001). Similar demographic parameters were observed between the quartiles except for age, which was older in the higher quartiles (median age of 3.4, 4.6, 4.4, and 6.2 years in quartiles 1, 2, 3, and 4, respectively (*p* < 0.001)). The majority of patients were males of Bedouin-Arab descent in all quartiles. Table 1 summarizes the background diseases with similar rates of prior steroid use, atopy, and different comorbidities between the quartiles.

Prematurity, neuromuscular disease, and metabolic disease were more common in the lower quartiles, while pulmonary hypertension was more common in the higher quartiles. Regarding chronic medical treatment, no significant difference was observed in the chronic anti-asthmatic treatment, indicating similar asthma control between the quartiles. Antiepileptic medications were used more commonly in the lower quartiles (Table 2). 

Table 3 summarizes the mean vital signs in the ED, with an elevated heart rate (131, 134, 154, and 137 beats per minute in quartiles 1, 2, 3 and 4, respectively, *p* = 0.01), higher temperature (37.71, 37.74, 39.55, and 37.4 Celsius in quartiles 1, 2, 3 and 4, respectively, *p* = 0.004), and higher respiratory rate (39, 43, 44 and 42 breaths per minute in the quartiles 1, 2, 3 and 4, respectively, *p* = 0.002) in the higher quartiles. The rate of tachypnea (adjusted to normal values for age), was correlated with higher quartiles with 85%, 89%, 94%, and 97% in quartiles 1, 2, 3, and 4, respectively (*p* < 0.001). Room air oxygen saturation was lower in the higher quartiles (93.5%, 93%, 92.1%, 92.5%, *p* < 0.001). 

^a^ SD—standard deviation. ^b^ Respiratory rate above the normal respiratory rate according to the American Heart Association. Bold values denote statistical significance at the *p* ≤ 0.05 level.

ED intervention characteristics are summarized in Table 4. ED urgency scale showed a decline across the quartiles, which correlated with higher mean clinical urgency (3.17, 2.72, 2.52, and 2.49 in quartiles 1, 2, 3, and 4, respectively, *p* < 0.001). Rates of supplemental oxygen, inhalation of anticholinergic agents, and intravenous magnesium sulfate were significantly higher in the higher quartiles. No significant difference was observed in chest X-ray findings among the quartiles. 

Data on admissions to the pediatric ward and PICU are presented in Table 5. Admission rate and length of stay (above 2 days) were significantly higher in the higher quartiles, with 66%, 76%, 79%, and 84% rates of admission in quartiles 1, 2, 3, and 4, respectively, (*p* < 0.001) and 35%, 36%, 42% and 48% for prolonged admission in quartiles 1, 2, 3 and 4, respectively (*p* = 0.034). The rate of PICU admissions was not statistically significant across the quartiles. 

Figure 1 present a multivariate analysis via generalized estimating equations (GEE) with a logistic distribution. Hospital admissions, a treatment in the resuscitation room (reflecting high urgency scale) in the ED, treatment with intravenous magnesium sulfate, prolonged hospitalization (≥two days), room air saturation < 92%, and tachypnea (normalized to age) showed an odds ratio of 2.9 (95% CI 1.79, 4.69, *p* = 0.001) for admission, 2.86 (95% CI 1.64, 5.0, *p* < 0.001) for treatment in the resuscitation room, 6.62 (95% CI 2.7, 16.2, *p* < 0.001) for intravenous magnesium sulfate treatment, 1.64 (95% CI 1.1, 2.45, *p* = 0.015) for prolonged hospitalization, 1.74 (95% CI 1.12, 2.7, *p* = 0.014) for room air saturation < 92% and 8 (95% CI 2.98, 21.5, *p* < 0.001) for tachypnea adjusted to age, in the higher quartile. 

## 4. Discussion 

The NLR, a blood count-derived parameter, is a marker of systemic inflammation, combining innate and allergic inflammation markers. It was previously suggested to be correlated with neutrophilic asthma [18], though its role in predicting the clinical outcome of asthmatic patients has not been elucidated yet. This study examined the correlation of the NLR with the clinical course of children who were presented with acute asthma exacerbation to the ED. Our results indicate that higher NLR is associated with a severe clinical course in children with acute exacerbations. Patients throughout the different quartiles had similar background diseases, chronic medical treatment, and asthma control. 

Patients in the higher NLR quartiles were presented with more severe respiratory distress than in the lower quartiles, as reflected by higher heart rate, respiratory rate, and lower room air oxygen saturation. This finding was not attributable to fever or age, as this group had a slightly higher mean age and lower body temperature. A significant correlation was found between the NLR and the need for treatment in the resuscitation room, based on the ED urgency scale. This correlation suggested once more that patients with higher NLR appeared more clinically deteriorated than others and required more intensive treatment. Treatment with oxygen and intra-venous magnesium sulfate was also correlated with increased NLR. 

Our results also demonstrated a higher hospitalization rate and length of stay with higher NLR. The risk for hospitalization for children within the highest NLR quartile was three times higher than in the lowest quartile, and the risk for prolonged hospitalization over two days equally increased. The PICU admission rate was twice as high among the higher quartiles, but this increase was not significant, possibly due to the small sample of children admitted to the PICU. 

Our findings support previous data regarding the inferior prognosis of neutrophilic asthma. Neutrophilic asthma is characterized by at least 65% neutrophils in the sputum of, or above, 5 × 10^6^ cells/mL [37]. Pathophysiologically, it is associated with activation of the Th17 pathway, IL-8 IL-1β, and TNFα. Recent data suggests it is correlated with decreasing FEV1 and resistance to steroid therapy [38]. To date, there are no specific therapeutical approaches to this asthma phenotype. Airway inflammation with dominant neutrophils can also be induced by respiratory tract infections, tobacco smoke exposure, and obesity. 

Our study results are in congruence with previous data regarding the association of NLR with the severity of exacerbation among asthmatic children. In a recent study from China, that examined 81 pediatric patients with asthma exacerbation, the severe exacerbation group (n = 10) had the highest levels of NLR (7.19), compared with the mild or moderate exacerbation groups, though the specific criteria for subgroup division are unclear. The optimal NLR cutoff level suggested was 1.723, with a sensitivity of 0.73 and specificity of 0.906 [28] .

A possible confounder influencing our results is a concurring bacterial infection which may elevate the neutrophil count. However, this seems unlikely in our cohort, due to the relatively lower body temperatures that characterized the highest NLR group and the similar use of antibiotics between the different groups as a surrogate marker for bacterial respiratory infection. Another possible influencing factor is previous steroid use, which was similar between the different groups in the week prior to their ED visit.

Another possible confounder is the physiologic distribution of NLR according to age and sex. All our groups presented similar sex distribution with a majority of male patients, and a slightly heterogenous age distribution. If we examine previous data on age-specific reference values, the pattern of change in the NLR values from birth to adolescence is mostly influenced by the highest peak in the neonatal and infancy period, which was not included in our study. Most of the childhood years are not characterized by substantial changes in the NLR [22]. 

Our study has a few limitations. First, is its retrospective nature. Our data was based on the documentation from the patient’s electronic files. All patients were diagnosed and treated in a single tertiary center, with a potentially excessive influence of the unique demographics in our region. Bedouin Arabs consist of up to 75% of our cohort. The combination of several characteristic factors, such as low socioeconomic status, poor sanitation, crowded houses, high rates of malnutrition, and smoking, all contribute to the previously described data on higher morbidity, specifically, respiratory infections, among the Bedouin pediatric population [39,40]. Another limitation is that we did not capture data regarding viral PCR in nasal swabs, which are common triggers for asthma exacerbation in children and could differ in blood count parameters and clinical presentations. NLR from blood samples prior to the current ED visits was out of the scope of this study, but it could be interesting to investigate the association of NLR as a chronic marker for asthma control and exacerbations. 

The strengths of our study are the relatively large population of children with a definition of asthma who were treated at the ED with anti-asthmatic treatments. In our study, we showed a significantly larger patient sample size. In addition, we examine multiple parameters of severity in order to demonstrate the potential clinical use of NLR in ED as an assessment tool for asthma exacerbation. We captured comprehensive ambulatory data on the patients, their blood tests, medication regimens, and radiology findings (that were interpreted by either a pediatric radiologist or a pulmonologist). Since the data was harvested from a single medical center, there is limited variability regarding admissions strategies and treatments, which strengthens the association between NLR and the severity measurements studied. 

In conclusion, the NLR is an objective tool that may have a role in assessing pediatric asthma exacerbation severity and the need for hospitalization. It is a feasible and safe measure, suited for the pediatric ED setting. NLR could potentially supply an additional decision tool that could assist the clinician in the identification of severe asthma cases that require admission and further inpatient care. However, further studies are warranted for its implementation in the clinical setting, providing validated range and cutoff values in which a high probability of severe asthma is likely. 

## Figures and Tables

**Table 1:** Demographic and background diseases, according to NLR ^a^ quartiles.

Characteristic	Quartile 1 N-207	Quartile 2 N-208	Quartile 3 N-208	Quartile 4 N-208	*p*-Value
NLR ^a^ Mean ± SD ^b^	1.6 ± 0.6	3.8 ± 0.7	6.8 ± 1.1	14.5 ± 5.5	**<0.001**
Age (years) Mean ± SD ^b^	5.3 ± 3.9	6.2 ± 4.1	6.1 ± 3.8	7.1 ± 3.8	**<0.001**
Male gender, n/N (%)	120/207 (58%)	129/208 (62%)	129/207 (62%)	114/208 (55%)	0.3
Bedouin ethnicity, n/N (%)	114/178 (64%)	123/181 (68%)	120/183 (66%)	129/171 (75%)	0.11
Steroid use before admission, n/N (%)	38/207 (18%)	37/208 (18%)	53/208 (25%)	48/208 (23%)	0.2
Prematurity (<36 + 6 weeks), n/N (%)	9/207 (4.3%)	2/208 (1.0%)	2/208 (1.0%)	1/208 (0.5%)	**0.013**
Bronchopulmonary dysplasia, n/N (%)	5/207 (2.4%)	2/208 (1.0%)	3/208 (1.4%)	0/208 (0%)	0.10
Pulmonary hypertension, n/N (%)	2/207 (1.0%)	0/208 (0%)	7/208 (3.4%)	5/208 (2.4%)	**0.023**
Developmental disease, n/N (%)	11/207 (5.3%)	7/208 (3.4%)	9/208 (4.3%)	4/208 (1.9%)	0.3
Neuromuscular disease, n/N (%)	9/207 (4.3%)	1/208 (0.5%)	2/208 (1.0%)	3/208 (1.4%)	**0.025**
Cardiac disease, n/N (%)	11/207 (5.3%)	7/208 (3.4%)	13/208 (6.2%)	6/208 (2.9%)	0.3
Metabolic disease, n/N (%)	4/207 (1.9%)	1/208 (0.5%)	0/208 (0%)	0/208 (0%)	**0.018**
Atopic dermatitis, n/N (%)	80/207 (39%)	95/208 (46%)	76/208 (37%)	81/208 (39%)	0.3
Allergic rhinitis, n/N (%)	5/207 (2.4%)	8/208 (3.8%)	3/208 (1.4%)	10/208 (4.8%)	0.2
Atopic diseases (combined), n/N (%)	81/207 (39%)	98/208 (47%)	77/208 (37%)	85/208 (41%)	0.2

^a^ Neutrophil-to-Lymphocyte ratio. ^b^ SD—standard deviation. Bold values denote statistical significance at the *p* ≤ 0.05 level.

**Table 2:** Chronic medication treatment, according to the quartiles.

Characteristic	Quartile 1 N-207	Quartile 2 N-208	Quartile 3 N-208	Quartile 4 N-208	*p*-Value
Inhaled corticosteroids, n/N (%)	75/207 (36%)	91/208 (44%)	79/208 (38%)	82/208 (39%)	0.4
Inhaled corticosteroids and long-acting beta-agonist, n/N (%)	16/207 (7.7%)	25/208 (12%)	22/208 (11%)	22/208 (11%)	0.5
Short-acting beta-agonist, n/N (%)	17/207 (8.2%)	14/208 (6.7%)	21/208 (10%)	17/208 (8.2%)	0.7
Leukotriene receptor antagonists, n/N (%)	31/207 (15%)	32/208 (15%)	22/208 (11%)	18/208 (8.7%)	0.10
Anticholinergics, n/N (%)	0/207 (0%)	1/208 (0.5%)	1/208 (0.5%)	0/208 (0%)	1
Antiepileptic medications, n/N (%)	22/207 (11%)	13/208 (6.2%)	10/208 (4.8%)	6/208 (2.9%)	**0.008**
Cardiovascular medications, n/N (%)	16/207 (7.7%)	7/208 (3.4%)	10/208 (4.8%)	5/208 (2.4%)	0.053
Antibiotics (chronic use), n/N (%)	6/207 (2.9%)	7/208 (3.4%)	13/208 (6.2%)	5/208 (2.4%)	0.2

Bold values denote statistical significance at the *p* ≤ 0.05 level.

**Table 3:** Measurements in the emergency department, according to quartiles.

Characteristic	Quartile 1 N-207	Quartile 2 N-208	Quartile 3 N-208	Quartile 4 N-208	*p*-Value
**Heart rate (beats/minute)** Mean ± SD ^a^ (N)	131 ± 21 (109)	135 ± 21 (107)	154 ± 146 (106)	137 ± 17 (99)	**0.010**
**Temperature (°C)** Mean ± SD ^a^ (N)	37.71 ± 0.95 (192)	37.74 ± 0.95 (176)	39.55 ± 24.99 (172)	37.40 ± 0.72 (165)	**0.004**
**Oxygen saturation (room air)** Mean ± SD ^a^ (N)	93.5 ± 7.1 (185)	93.0 ± 5.6 (179)	92.1 ± 5.5 (173)	92.5 ± 4.6 (176)	**<0.001**
**Room air oxygen saturation < 92%**	60/185 (32%)	75/179 (42%)	79/173 (46%)	75/176 (43%)	0.061
**Systolic blood pressure (mm Hg)** Mean ± SD ^a^ (N)	107 ± 11 (178)	108 ± 11 (162)	109 ± 11 (160)	111 ± 11 (171)	**0.002**
**Diastolic blood pressure (mm Hg)** Mean ± SD ^a^ (N)	66 ± 10 (178)	66 ± 9 (162)	66 ± 10 (160)	66 ± 9 (171)	1
**Respiratory rate (breaths/minute)** Mean ± SD ^a^ (N)	39 ± 13 (196)	43 ± 15 (185)	44 ± 13 (179)	42 ± 12 (183)	**0.002**
**Tachypnea** (respiratory rate according to age) ^b^ n/N (%)	167/196 (85%)	165/185 (89%)	169/179 (94%)	178/183 (97%)	**<0.001**

**Table 4:** Treatment and interventions in the emergency department, according to the quartiles.

Characteristic	Quartile 1 N-207	Quartile 2 N-208	Quartile 3 N-208	Quartile 4 N-208	*p*-Value
ED ^a^ Urgency ^1^, Median (IQR ^b^)	3.00 (3.00, 4.00)	3.00 (2.00, 3.25)	2.50 (1.00, 3.00)	3.00 (1.00, 3.00)	**<0.001**
Oxygen, n/N (%)	69/207 (33%)	98/208 (47%)	92/208 (44%)	101/208 (49%)	**0.007**
Inhalation of Beta-agonists, n/N (%)	125/207 (60%)	137/208 (66%)	134/208 (64%)	139/208 (67%)	0.5
Inhalation of Anticholinergic, n/N (%)	91/207 (44%)	104/208 (50%)	109/208 (52%)	124/208 (60%)	**0.015**
Inhaled corticosteroids, n/N (%)	9/207 (4.3%)	12/208 (5.8%)	15/208 (7.2%)	13/208 (6.2%)	0.7
Systemic Steroids PO, n/N (%)	82/207 (40%)	71/208 (34%)	62/208 (30%)	57/208 (27%)	**0.043**
Systemic Steroids IV, n/N (%)	18/207 (8.7%)	25/208 (12%)	23/208 (11%)	27/208 (13%)	0.5
Antihistamines, n/N (%)	1/207 (0.5%)	0/208 (0%)	0/208 (0%)	0/208 (0%)	0.2
Antibiotics, n/N (%)	10/207 (4.8%)	9/208 (4.3%)	6/208 (2.9%)	10/208 (4.8%)	0.7
Sodium chloride Inhalation, n/N (%)	113/207 (55%)	121/208 (58%)	127/208 (61%)	132/208 (63%)	0.3
Intravenous Magnesium Sulphate, n/N (%)	6/207 (2.9%)	20/208 (9.6%)	26/208 (12%)	32/208 (15%)	**<0.001**
Findings on chest X-ray, n/N (%) Normal Hyperinflation Infiltrate Atelectasis	37/63 (59%) 8/63 (13%) 12/63 (19%) 6/63 (9.5%)	43/82 (52%) 13/82 (16%) 16/82 (20%) 10/82 (12%)	45/91 (49%) 19/91 (21%) 12/91 (13%) 15/91 (16%)	54/95 (57%) 13/95 (14%) 17/95 (18%) 11/95 (12%)	0.8

^a^ ED—Emergency department. ^b^ IQR—interquartile range. ^1^ Allon et al. [29]. Bold values denote statistical significance at the *p* ≤ 0.05 level.

**Table 5:** Admissions and hospitalizations data, according to quartiles.

Characteristic	Quartile 1 N-207	Quartile N-208	Quartile 3 N-208	Quartile 4 N-208	*p*-Value
**Hospitalization,** n/N (%)	137/207 (66%)	158/208 (76%)	165/208 (79%)	175/208 (84%)	**<0.001**
LOS *, Median (IQR)	2.00 (1.00, 3.00)	1.00 (1.00, 2.00)	2.00 (1.00, 2.00)	2.00 (1.00, 3.00)	0.2
LOS * ≥ 2, n/N (%)	73/207 (35%)	75/208 (36%)	88/208 (42%)	99/208 (48%)	**0.034**
**PICU** **, n/N (%)	5/207 (2.4%)	10/208 (4.8%)	12/208 (5.8%)	12/208 (5.8%)	0.3

* LOS—length of stay in the hospital, ** PICU—pediatric intensive care unit. Bold values denote statistical significance at the *p* ≤ 0.05 level.

## Data Availability

The database used and/or analyzed during the current study are available from the corresponding author on reasonable request.

## Supplementary Materials

The following supporting information can be downloaded at: https://www.mdpi.com/article/10.3390/jcm12093312/s1, Table S1: Several generalized estimating equations (GEE), with a logistic distribution, for the following variables: hospital admissions, the need for a resuscitation room, intravenous magnesium sulfate, admission ≥ two days, room air saturation < 92%, and tachypnea according to age.

## Abbreviations

NLR	neutrophil-to-lymphocyte ratio
ED	emergency department
IQR	interquartile range
LOS	length of stay in the hospital
PICU	pediatric intensive care unit
