# Supplementary material for: Elevated Neutrophil-to-Lymphocyte Ratio Is Associated with Severe Asthma Exacerbation in Children
Source: J Clin Med. 2023 May 6;12(9):3312. doi: 10.3390/jcm12093312 (PMC10179107; doi:10.3390/jcm12093312)
Supplement: Supplementary file 1 [file jcm-12-03312-s001.zip › jcm-2364286-supplementary.pdf]

Table S1

Multivariate GEE* logistic regression – **Hospital admissions** as a dependent variable

**Characteristic**	**OR**[1]	**95% CI**[1]	**p-value**
**NLR quartile**			
**0% - 24.9%**	—	—	
**25% - 49.9%**	1.67	1.08, 2.57	**0.020**
**50% - 74.9%**	2.02	1.30, 3.13	**0.002**
**75% - 100%**	2.90	1.79, 4.69	**<0.001**
**Age**	0.97	0.93, 1.01	0.11

[1] OR = Odds Ratio, CI = Confidence Interval

Multivariate GEE* logistic regression – **Resuscitation room** admission as a dependent variable

Characteristic	OR[1]	95% CI[1]	p-value
**NLR quartile**			
**0% - 24.9%**	—	—	
**25% - 49.9%**	2.30	1.32, 4.02	**0.003**
**50% - 74.9%**	2.85	1.65, 4.92	**<0.001**
**75% - 100%**	2.86	1.64, 5.00	**<0.001**
**Age**	1.00	0.96, 1.05	0.9

[1] OR = Odds Ratio, CI = Confidence Interval

Multivariate GEE* logistic regression – **Intravenous Magnesium treatment** as a dependent variable

**Characteristic**	**OR**[1]	**95% CI**[1]	**p-value**
**NLR quartile**			
**0% - 24.9%**	—	—	
**25% - 49.9%**	3.70	1.46, 9.42	**0.006**
**50% - 74.9%**	4.97	2.00, 12.4	**<0.001**
**75% - 100%**	6.62	2.70, 16.2	**<0.001**
**Age**	0.96	0.91, 1.01	0.13

[1] OR = Odds Ratio, CI = Confidence Interval

Multivariate GEE* logistic regression – **Admission ≥ two days** as a dependent variable

**Characteristic**	**OR**[1]	**95% CI**[1]	**p-value**
**NLR quartile**			
**0% - 24.9%**	—	—	
**25% - 49.9%**	1.03	0.69, 1.54	0.9
**50% - 74.9%**	1.34	0.90, 1.99	0.2
**75% - 100%**	1.64	1.10, 2.45	**0.015**
**Age**	1.01	0.97, 1.04	0.7

[1] OR = Odds Ratio, CI = Confidence Interval

Multivariate GEE* logistic regression – **Room air saturation < 92 %** as a dependent variable

Characteristic	OR[1]	95% CI[1]	p-value
**NLR quartile**			
**0% - 24.9%**	—	—	
**25% - 49.9%**	1.58	1.03, 2.44	**0.038**
**50% - 74.9%**	1.85	1.20, 2.86	**0.005**
**75% - 100%**	1.74	1.12, 2.70	**0.014**
**Age**	0.95	0.91, 0.99	**0.010**
**[1] OR = Odds Ratio, CI = Confidence Interval**			

Multivariate GEE* logistic regression – **Tachypnea according to age** as a dependent variable

Characteristic	OR[1]	95% CI[1]	p-value
**NLR quartile**			
**0% - 24.9%**	—	—	
**25% - 49.9%**	1.61	0.85, 3.05	0.15
**50% - 74.9%**	3.28	1.56, 6.88	**0.002**
**75% - 100%**	8.00	2.98, 21.5	**<0.001**
**Age**	0.89	0.84, 0.94	**<0.001**
**[1] OR = Odds Ratio, CI = Confidence Interval**			

*GEE- Generalized estimating equation
